# Supplementary material for: Drivers and impact of the early silent invasion of SARS-CoV-2 Alpha
Source: Nat Commun. 2024 Mar 9;15:2152. doi: 10.1038/s41467-024-46345-1 (PMC10925057; doi:10.1038/s41467-024-46345-1)
Supplement: Supplementary file 1 — Supplementary Information [file 41467_2024_46345_MOESM1_ESM.pdf]

Supplementary Information

Drivers and impact of the early silent invasion of SARS-CoV-2 Alpha

Benjamin Faucher[1], Chiara E. Sabbatini[1], Peter Czuppon[2], Moritz U.G. Kraemer[3,4], Philippe Lemey[5], Vittoria Colizza[†1,7], Francois Blanquart[†6], Pierre-Yves Boëlle[†1], Chiara Poletto[†*8]

[1]Sorbonne Université, INSERM, Institut Pierre Louis d'Epidémiologie et de Santé Publique (IPLESP), F75012, France

[2]Institute for Evolution and Biodiversity, University of Münster, Münster 48149, Germany

[3]Department of Biology, University of Oxford, Oxford, UK

[4]Pandemic Sciences Institute, University of Oxford, UK

[5]Department of Microbiology, Immunology and Transplantation, Rega Institute, Laboratory for Clinical and Epidemiological Virology, KU Leuven, Leuven, Belgium

[6]Center for Interdisciplinary Research in Biology, CNRS, Collège de France, PSL Research University, Paris 75005, France

[7]Department of Biology, Georgetown University, Washington, District of Columbia, USA

[8]Department of Molecular Medicine, University of Padova, 35121 Padova, Italy

*Corresponding author. Email : chiara.poletto@unipd.it

[†]These authors contributed equally to this work

This PDF file includes :

- Supporting text
- Supplementary Fig. 1 to 5
- Supplementary Tables 1 to 5
- SI References

Supporting information Text

Arrival times, number of passengers and sequencing coverage

Assuming exponential increase of cases in the origin country, the time of first arrival with passenger flow p is Gumbel distributed with mean proportional to $log\ (p)$ [1]. Assuming collection coverage s on top, the mean arrival time based on first collection scales as $log\ (p)\ + log\ (s)$ and "iso-arrival-time" lines are as $log\ (p)\ + log\ (s)\ =\ k$ (anti diagonals). We report such lines in Fig. 1B as reference.

International dissemination model: Sensitivity analysis

In the sensitivity analysis we tested the following assumptions:

- Mean incubation period equal to 4 days
- Mean incubation period equal to 6 days
- Delays computed for each countries after the alert averaged over a sliding time window of 7 days
- Percentage of case detection outside the UK, K_c= 25%
- Flights from all England airports with a catchment population of 56 millions inhabitants
- No changepoint for the Alpha incidence exponential growth in the UK
- Two changepoints for the Alpha incidence exponential growth in the UK an 5 Nov 2020 and 2 Dec 2020

Supplementary Table 5 shows the results of the sensitivity analysis. For the baseline scenario and the sensitivity models tested we provide best estimates and some model predictions chosen as reference. Varying the parameters had little impact on the parameters estimated in the model. The number of countries with introduction before 31 Dec 2020 increased in the

following cases: delays from collection to submission for Alpha computed for each country aggregated over 7 days, 25% detection of imported cases, no change of slope.

Local dynamics in the USA at a finer spatial scale

The analysis of Alpha local spread for the USA shows that this country is out of trend, with a high number of predicted Alpha cases compared with model estimates. Here, we carry out the comparison for two individual states, California and Florida, and for New York City - see sources of data reported in Supplementary Table 2. These locations were the port of entry of Alpha into the US, with early reported Alpha cases linked directly to the UK [2,3].

As for the USA as a whole, the autochthonous model A was fed with importation fluxes estimated from the international dissemination model and we compared the model-predicted number of Alpha cases with the empirical estimates. We present these results on Supplementary Fig. 4.

For California and New York City, the comparison between model and empirical estimates follows a trend similar to European countries. Florida registered a high proportion of Alpha cases [2]. Such a high level of Alpha circulation can be compatible with model predictions in a scenario of early Alpha introduction, i.e. introduction dates close to the lower bound of the range predicted by the model.

Median seeding time

In the reference case in which traveling fluxes are constant in time and R_t is the same in the destination country as in the UK, the reference median date of seeding would fall halfway between the date of emergence and 31 Dec 2020 (see Methods). The median seeding dates of active chains at the end of 2020 departed from this assumed scenario. In Fig. 4D, we found that there was a negative correlation between the overall reproduction ratio over the period and the difference between computed median seeding date and reference median seeding date (spearman correlation -0.81 with p-value = 0.049), implying that lower transmissibility overall led to less success in early introductions. In Supplementary Fig. 5 we show that there was no significant correlation between the international traffic drop and the difference of the median seeding date with the reference date.

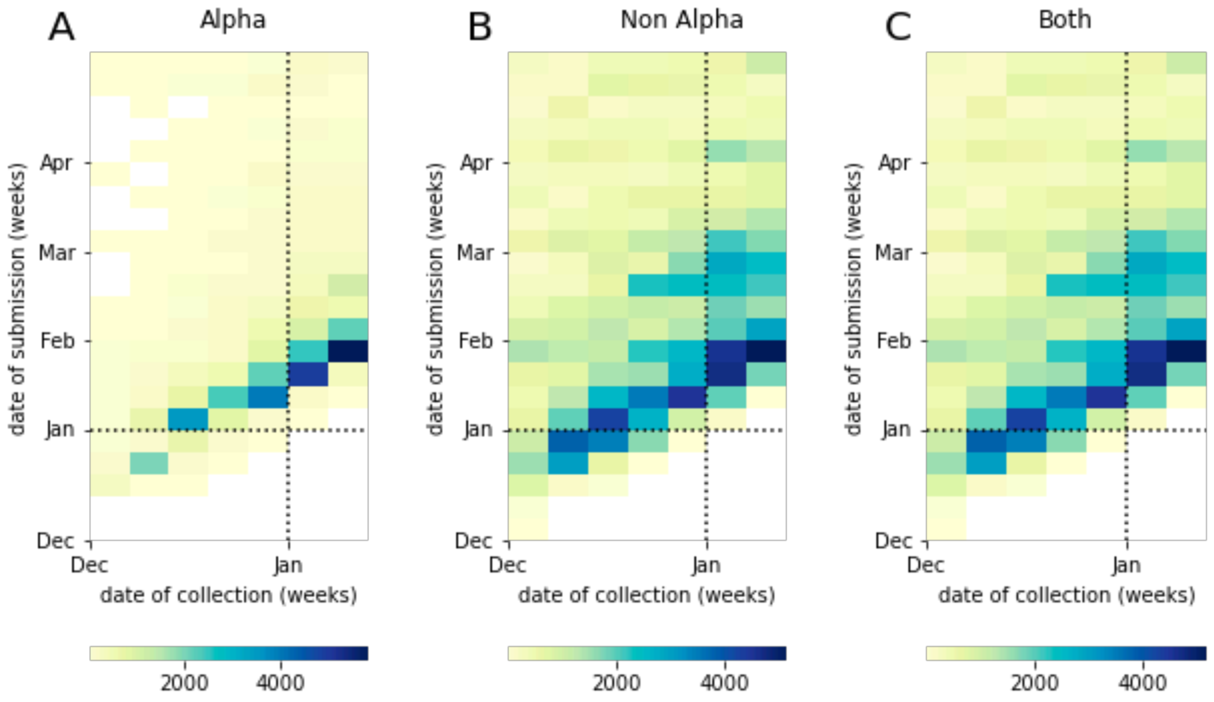

Supplementary Fig 1. Occurrences of delays between collection and submission in time. (**A**) Alpha variant.(**B**) Non Alpha variants. (**C**) Both.

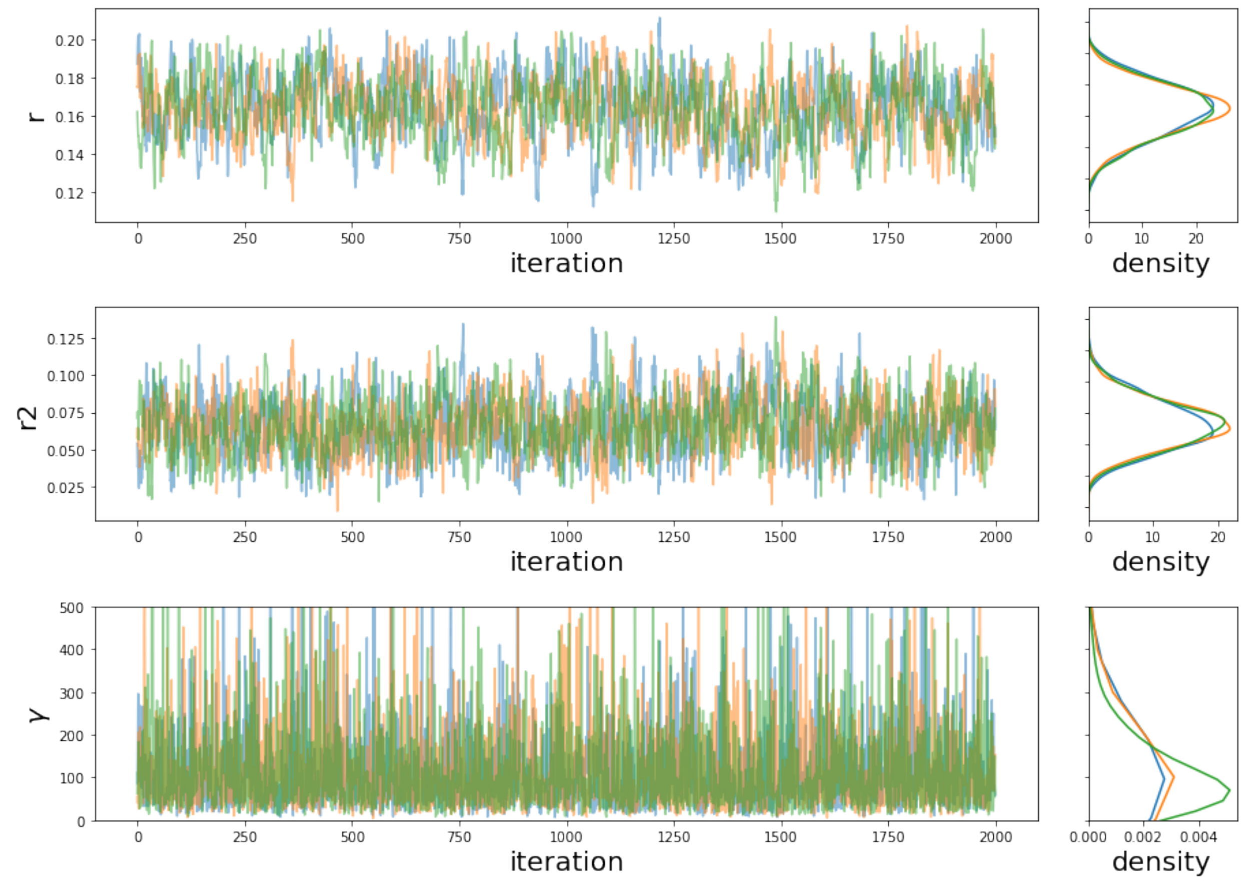

Supplementary Fig. 2. MCMC convergence plot. The fitted model has 2 exponential growth rates (r_1,r_2) with a changepoint on November 5th,2020. Three independent chains (red, green,blue) were run for 100000 iterations, with 50000 discarded as burn-in. Posterior samples were thinned 1 in 25.

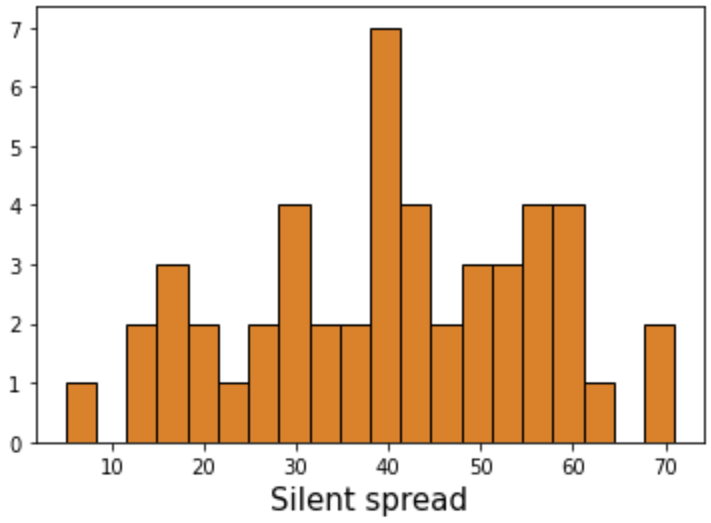

Supplementary Fig. 3 Distribution of silent spread in days (n=69). Silent spread is computed as in Fig. 3D.

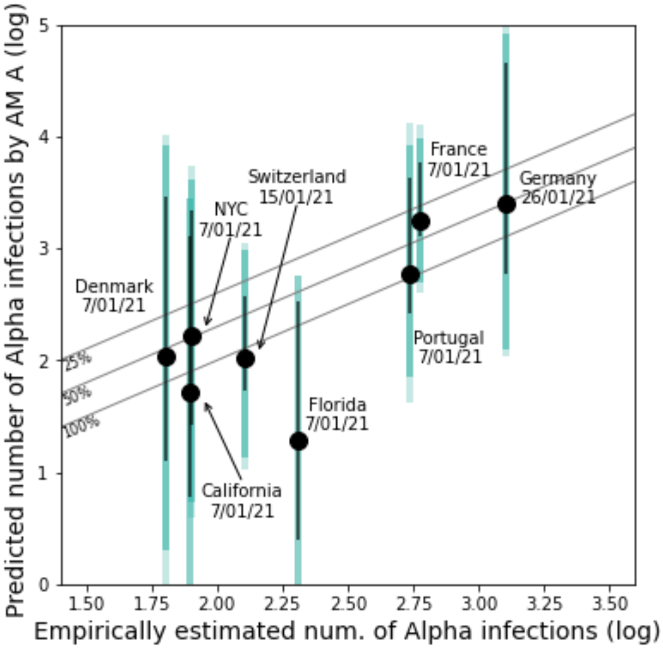

Supplementary Fig. 4. Model vs. empirical cases of Alpha as in Fig. 4 of the main paper. Here, the USA is replaced by California, New York City (NYC) and Florida to address spatial heterogeneity inside the USA. The empirical estimates of Alpha cases are computed by multiplying the Alpha frequency from virological investigations by the reported COVID-19 incidence at the same date - the date is indicated in the plot. Model estimates are obtained with the autochthonous model A (AM A in the plot). Gray lines show ratios of 100%, 50% and 25% between observed and predicted infections attributable to reporting. Black error bars indicate the prediction interval over 500 stochastic simulations obtained with the median volume of Alpha introduction, output of the international dissemination model assuming a 7-day delay between case and infection. Dark colored bars account for the variability in the output of the autochthonous model accounting for the upper and the lower limit of the prediction interval of the Alpha introductions as given by the international dissemination model. Light colored bars account for variability in the delay from infection to case reporting (ranging from 4 days to 10 days).

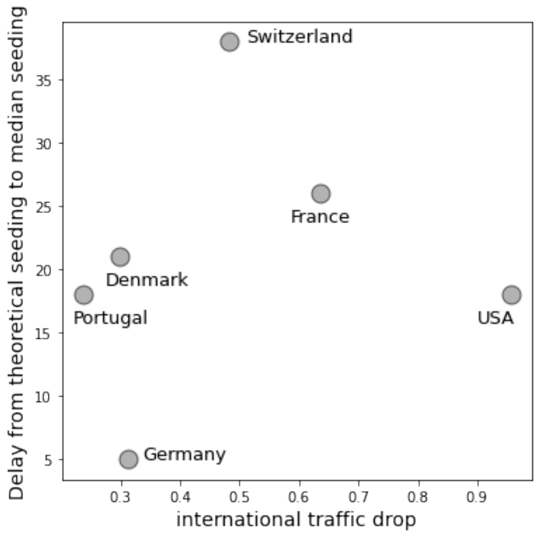

Supplementary Fig. 5. Difference between the median seeding date predicted by the autochthonous model A with the same quantity when R_t is the same in all countries and traveling fluxes do not change in time, plotted against the international traffic drop. The international traffic drop is computed as the international traffic in Nov 2020 divided by the average of international traffic between Sep 2020 and Oct 2020. The Spearman correlation coefficient does not show a correlation between the two quantities (coefficient = 0.23, p-value = 0.66, t-distribution with n-2 dof).

Supplementary tables

Country	Date of cases surveyed for Alpha	Daily number cases	Frequency Alpha	Computed number of Alpha infections	Source
France	7 Jan 2021	18,004	0.033	594	[4]
Portugal	4-10 Jan 2021	8,062 (7/01/2021)	0.068*	548	[5]
Germany	23-29 Jan 2021	12,370 (26/01/2021)	0.103	1,274	[6]
Denmark	4-10 Jan 2021	1,825 (7/01/2021)	0.035	64	[7]
Switzerland	15 Jan 2021	2,204	0.058	128	[8]
USA	7 Jan 2021	248,566	0.0048	1,193	[2]

Supplementary Table 1. Summary of data used for the validation. France epidemiological report last accessed 26/05/2023. Germany report last accessed 26/05/2023. Denmark website last accessed 26/05/2023 (2 Mar 2021 version).

* At week 01 of 2021, 7.38% of cases were suspicions of Alpha, 92% of which are true Alpha.

Country	Date of cases surveyed for Alpha	Daily number cases	Frequency Alpha	Computed number of Alpha infections	Source
California	7 Jan 2021	43314	0.0018	78	[2]
Florida	7 Jan 2021	15939	0.0128	204	[2]
NYC	7 Jan 2021	5808	0.013738	80	[9]

Supplementary Table 2. Summary of data used for the local spread analysis for the locations within the US explored in Supplementary Fig. 4.

Parameter	Description	Baseline value
K_{UK}	Fraction of sampled Covid cases.	0.25
K_c	Fraction of sampled imported Covid cases.	0.5
e	Incubation period.	5 days
N	Population in the catchment area of London airports	36M
T_0	Beginning of the risk window for VOC emergence in the UK	15 Aug 2020
$Tcp2$	Date of change of the exponential transmission growth in the UK.	5 Nov 2020

Supplementary Table 3. Summary of the parameters values assumed in the international dissemination model.

Parameter	Description	Prior distribution
r_1	Exponential growth rate in UK up to 5 Nov 2020	Exp(0.1)
r_2	Exponential growth rate in UK after 5 Nov 2020	N(0,1)
γ	Ratio of sampling among traveling vs. non-traveling cases after 18 Dec 2020	Exp(0.01)

Supplementary Table 4. Summary of the estimated parameters and their prior distribution.

Scenario	r_1	r_2	γ	Predicted time of emergence in the UK	Median date of first introduction for France, and Denmark	#countries with introduction before 31 Dec 2020
Baseline	0.17 [0.14;0.20]	0.055 [0.02;0.097]	51.67 [12.43;310.11]	08/09 [21/08;19/09]	17/10 [18/09-03/11] - 05/11 [08/10-06/12]	65 [52-73]
Incubation = 4 days	0.17 [0.14;0.20]	0.056 [0.024;0.096]	51.51 [11.72;307.19]	09/09 [21/08;18/09]	17/10 [20/09-03/11] - 05/11 [09/10-07/12]	65 [52-73]
Incubation = 6 days	0.17 [0.14;0.20]	0.056 [0.024;0.097]	52.36 [11.89;301.70]	06/09 [19/08;16/09]	17/10 [19/09-03/11] - 05/11 [09/10-06/12]	65 [52-73]
Delays computed by country, aggregated on 7 days After 18 Dec 2020	0.16 [0.11;0.19]	0.086 [0.043;0.13]	36.26 [7.60;186.32]	02/09 [17/08;18/09]	17/10 [20/09-09/11] - 09/11 [09/10-07/12]	69 [60-73]
25% detection of imported cases	0.18 [0.15;0.21]	0.058 [0.025;0.1]	51.20 [11.53;300.63]	06/09 [19/08;18/09]	15/10 [19/09-31/10] - 31/10 [07/10-30/11]	70 [61-73]
Air travel : flight from England	0.17 [0.14;0.20]	0.056 [0.025;0.097]	73.13 [15.46;415.13]	02/09 [17/08;18/09]	19/10 [21/09-06/11] - 07/11 [10/10-08/12]	64 [47-72]
No change of slope	0.12 [0.11;0.13]		22.46 [6.75;94.21]	30/08 [16/08;12/09]	23/10 [14/09-13/11] - 24/11 [12/10-13/12]	69 [59-73]
2 changes of slope : 11/5 and 12/2	0.16 [0.12;0.20]	r2 : 0.07 [0.026,0.15] r3 : 0.023 [0.022,0.48]	61.32 [13.19;332.14]	02/09 [17/08;17/09]	18/10 [18/09-08/10] - 08/11 [09/10-07/12]	65 [51-73]

Supplementary Table 5. Sensitivity analysis of the international dissemination model.

References

1. Gautreau, A., Barrat, A. & Barthélemy, M. Global disease spread: Statistics and estimation of arrival times. *Journal of Theoretical Biology* **251**, 509–522 (2008).
2. Washington, N. L. *et al.* Emergence and rapid transmission of SARS-CoV-2 B.1.1.7 in the United States. *Cell* **184**, 2587-2594.e7 (2021).
3. Alpert, T. *et al.* Early introductions and transmission of SARS-CoV-2 variant B.1.1.7 in the United States. *Cell* **184**, 2595-2604.e13 (2021).
4. SPF. COVID-19 : point épidémiologique du 28 janvier 2021. https://www.santepubliquefrance.fr/maladies-et-traumatismes/maladies-et-infections-respiratoires/infection-a-coronavirus/documents/bulletin-national/covid-19-point-epidemiologique-du-28-janvier-2021.
5. Borges, V. *et al.* Tracking SARS-CoV-2 lineage B.1.1.7 dissemination: insights from nationwide spike gene target failure (SGTF) and spike gene late detection (SGTL) data, Portugal, week 49 2020 to week 3 2021. *Eurosurveillance* **26**, 2100131 (2021).
6. RKI - Coronavirus SARS-CoV-2 - Berichte zu Virusvarianten von SARS-CoV-2 in Deutschland. https://www.rki.de/DE/Content/InfAZ/N/Neuartiges_Coronavirus/DESH/Berichte-VOC-tab.html.
7. Danish Covid-19 Genome Consortium. https://covid19genomics.dk/statistics.
8. Chen, C. *et al.* Quantification of the spread of SARS-CoV-2 variant B.1.1.7 in Switzerland. *Epidemics* **37**, 100480 (2021).
9. coronavirus-data/variants at master · nychealth/coronavirus-data. *GitHub* https://github.com/nychealth/coronavirus-data/tree/master/variants.
