# Supplementary material for: Drivers and impact of the early silent invasion of SARS-CoV-2 Alpha
Source: Nat Commun. 2024 Mar 9;15:2152. doi: 10.1038/s41467-024-46345-1 (PMC10925057; doi:10.1038/s41467-024-46345-1)
Supplement: Supplementary file 3 — Reporting Summary [file 41467_2024_46345_MOESM3_ESM.pdf]

nature portfolio

Corresponding author(s): Chiara Poletto
Last updated by author(s): 15/2/2024

Reporting Summary

Nature Portfolio wishes to improve the reproducibility of the work that we publish. This form provides structure for consistency and transparency in reporting. For further information on Nature Portfolio policies, see our Editorial Policies and the Editorial Policy Checklist.

Statistics

For all statistical analyses, confirm that the following items are present in the figure legend, table legend, main text, or Methods section.

n/a	Confirmed	
☐	☒	The exact sample size (*n*) for each experimental group/condition, given as a discrete number and unit of measurement
☒	☐	A statement on whether measurements were taken from distinct samples or whether the same sample was measured repeatedly
☐	☒	The statistical test(s) used AND whether they are one- or two-sided<br>*Only common tests should be described solely by name; describe more complex techniques in the Methods section.*
☐	☒	A description of all covariates tested
☒	☐	A description of any assumptions or corrections, such as tests of normality and adjustment for multiple comparisons
☐	☒	A full description of the statistical parameters including central tendency (e.g. means) or other basic estimates (e.g. regression coefficient) AND variation (e.g. standard deviation) or associated estimates of uncertainty (e.g. confidence intervals)
☐	☒	For null hypothesis testing, the test statistic (e.g. *F*, *t*, *r*) with confidence intervals, effect sizes, degrees of freedom and *P* value noted<br>*Give P values as exact values whenever suitable.*
☐	☒	For Bayesian analysis, information on the choice of priors and Markov chain Monte Carlo settings
☒	☐	For hierarchical and complex designs, identification of the appropriate level for tests and full reporting of outcomes
☒	☐	Estimates of effect sizes (e.g. Cohen's *d*, Pearson's *r*), indicating how they were calculated

Our web collection on statistics for biologists contains articles on many of the points above.

Software and code

Policy information about availability of computer code

Data collection	No software was used for the datacollection
Data analysis	Analysis was done using custom code in R (v4.3), C++ (v11), and Python 3.8.5.<br>All code was packaged in the "importFromUK" package available from the GitHub : https://github.com/BenjaminFaucher/Alpha-dissemination

For manuscripts utilizing custom algorithms or software that are central to the research but not yet described in published literature, software must be made available to editors and reviewers. We strongly encourage code deposition in a community repository (e.g. GitHub). See the Nature Portfolio guidelines for submitting code & software for further information.

Data

Policy information about availability of data

All manuscripts must include a data availability statement. This statement should provide the following information, where applicable:

- Accession codes, unique identifiers, or web links for publicly available datasets
- A description of any restrictions on data availability
- For clinical datasets or third party data, please ensure that the statement adheres to our policy

The findings of this study are based on metadata associated with a total of 1,735,675 sequences available on GISAID and submitted between 15 Aug 2020 and 1 Jun 2021 included and downloaded on 2 Jun 2021 via gisaid.org (GISAID: EPI_SET_230724tv). To view the contributors of each sequence associated with the metadata we used, visit https://doi.org/10.55876/gis8.230724tv. Proprietary airline data are commercially available from OAG and IATA databases (https://www.iata.org/). All

other data used in the study are publicly available online. Channel Tunnel data were obtained from https://www.eurotunnelfreight.com/fr/2021/01/trafic-navettes-du-mois-de-decembre-2020/, ferries data were obtained from https://www.gov.uk/government/statistical-data-sets/sea-passenger-statistics-spas, COVID-19 cases were obtained from https://github.com/CSSEGISandData/COVID-19, COVID-19 deaths were obtained from https://www.ecdc.europa.eu/en/publications-data/data-national-14-day-notification-rate-covid-19, Alpha cases in the UK were obtained from https://assets.publishing.service.gov.uk/media/6059e4ad8fa8f545d5c339fc/Variants_of_Concern_VOC_Technical_Briefing_7_England.pdf.

Research involving human participants, their data, or biological material

Policy information about studies with human participants or human data. See also policy information about sex, gender (identity/presentation), and sexual orientation and race, ethnicity and racism.

Reporting on sex and gender	Our study does not involve human research participants
Reporting on race, ethnicity, or other socially relevant groupings	Our study does not involve human research participants
Population characteristics	Our study does not involve human research participants
Recruitment	Our study does not involve human research participants
Ethics oversight	Our study does not involve human research participants

Note that full information on the approval of the study protocol must also be provided in the manuscript.

Field-specific reporting

Please select the one below that is the best fit for your research. If you are not sure, read the appropriate sections before making your selection.

☒ Life sciences ☐ Behavioural & social sciences ☐ Ecological, evolutionary & environmental sciences

For a reference copy of the document with all sections, see nature.com/documents/nr-reporting-summary-flat.pdf

Life sciences study design

All studies must disclose on these points even when the disclosure is negative.

Sample size	Analyses of Figures 2 and 3 were based on posterior sample of size 2000. Analyses of Figure 4 were based on 500 independent stochastic simulations. These number of simulations guarantee that the coefficient of variation in estimated variances is less than 5% (normal approximation, CV ~ sqrt(2/n) for variance).
Data exclusions	All countries that submitted sequences on GISAID in the period Sept 2020 - Dec 2020 were considered in the study, as detailed in the main paper.
Replication	Data imputation and Markov Chain Monte Carlo were run several times with similar results. For the autochthonous models A and B, each simulation was executed independently and with a different seed of the random number generator. Analyses of Figures 2 and 3 were based on posterior sample of size 2000. Analyses of Figure 4 were based on 500 independent stochastic simulations.
Randomization	Since this is a computational modeling study, randomization procedures were not applicable. Precisely, the use of randomization was not relevant since we do not perform statistical tests in experimental settings but data modelling of observationnal data.
Blinding	Not relevant since the conclusions are not based on subjective measurements or comparisons that could be biased by human interventions.

Reporting for specific materials, systems and methods

We require information from authors about some types of materials, experimental systems and methods used in many studies. Here, indicate whether each material, system or method listed is relevant to your study. If you are not sure if a list item applies to your research, read the appropriate section before selecting a response.

Materials & experimental systems

n/a	Involved in the study
☒	☐ Antibodies
☒	☐ Eukaryotic cell lines
☒	☐ Palaeontology and archaeology
☒	☐ Animals and other organisms
☒	☐ Clinical data
☒	☐ Dual use research of concern
☒	☐ Plants

Methods

n/a	Involved in the study
☒	☐ ChIP-seq
☒	☐ Flow cytometry
☒	☐ MRI-based neuroimaging
